# Effects of Native Arbuscular Mycorrhizae Isolated on Root Biomass and Secondary Metabolites of *Salvia miltiorrhiza* Bge

**DOI:** 10.3389/fpls.2021.617892

**Published:** 2021-02-02

**Authors:** Yan-Hong Wu, Hai Wang, Min Liu, Bo Li, Xin Chen, Yun-Tong Ma, Zhu-Yun Yan

**Affiliations:** State Key Laboratory of Characteristic Chinese Medicine Resources in Southwest China, School of Pharmacy, Chengdu University of Traditional Chinese Medicine, Chengdu, China

**Keywords:** *Salvia miltiorrhiza*, arbuscular mycorrhizal fungi, biomass, phenolic acids, tanshinones, quality

## Abstract

Arbuscular mycorrhiza fungi (AMFs) are a group of soil-dwelling fungi that form symbiotic associations with plants, to mediate the secondary metabolism and production of active ingredients in aromatic and medicinal plants. Currently, there is little research on *Salvia miltiorrhiza* Bge. inoculation with native AMFs and the concomitant effects on growth and secondary metabolites. In this study, *S. miltiorrhiza* was treated with eight AMFs, i.e., *Glomus formosanum*; *Gl. tenebrosum*; *Septoglomus constrictum*; *Funneliformis geosporum*; *Rhizophagus manihotis*; *Ambispora gerdemanii*; *Acaulospora laevis*; *Ac. tuberculata*, to investigate the influence of AMF inoculation on biomass and secondary production under greenhouse conditions in *S. miltiorrhiza* roots. The results showed that mycorrhiza formation rates were between 54.83 and 86.10%. Apart from *Ac. laevis* and *Gl. tenebrosum* treatment, the roots biomass of the other treatment groups was effectively increased, and the fresh and dry weight of the plant inoculated with *Fu. geosporum* were increased by 86.76 and 86.95%, respectively. Specifically, AMF treatments also impacted on phenolic acids production; inoculation with both *Fu. geosporum* or *Ac. laevis* significantly reduced total phenolic acids, whereas the other treatments effectively increased these levels, of which *Gl. formosanum* generated significant levels. Most AMF-plant symbiotic experiments facilitated phenolic acid accumulation in the secondary metabolites of *S. miltiorrhiza* (except *Ac. laevis*). This study showed that most native AMFs inoculation with *S. miltiorrhiza* promoted roots growth and increased secondary metabolites production (especially phenolic acids). Going forward, inoculation of native AMF is a promising method to improve the quality and yield of *S. miltiorrhiza* and should be considered during production.

## Introduction

As a kind of quite important medicinal plant in China, and, to a lesser extent, in Japan and the United States, *Salvia miltiorrhiza* Bge. (Danshen or Red sage, *Lamiaceae*,) was extensively used to treat menstrual disorders, and cardiovascular, and cerebrovascular disease for many years (Chun-Yan et al., 2015; Wang et al., 2017; Ren et al., 2019; Wu et al., 2020). These therapeutic effects are attributed to intrinsic water-soluble phenolic acids and lipophilic diterpenoid quinones (Wang et al., 2018). Previous studies have shown that there were 27 species of arbuscular mycorrhiza fungi (AMF) in eight genera existed in the rhizosphere soil of *S.miltiorrhiza* in China (Liu et al., 2017). *S. miltiorrhiza* was easily colonized with AMF species, such as *Glomus*, *Acaulospora*, *Scutellospora*, and *Entrophospora*, to generate good colonization rates (Wang and He, 2009; He et al., 2010). AMFs affected the content and composition of secondary metabolites, e.g., polyphenols, flavonoids, carotenoids, and phytoestrogens, both in crop and medicinal plants (Zeng et al., 2013; Sbrana et al., 2014; Pedone-Bonfim et al., 2015; Bruisson et al., 2016). However, the effects of these native AMFs on the yield and quality of *S. miltiorrhiza* have not been systematically understood.

AMFs are essential elements of the agricultural ecosystem (Thirkell et al., 2017) and form mutualistic symbioses with approximately 80% of land plant species (Smith et al., 2010). The extensive AMFs mycelium facilitates nutrient mobility in soils (Bücking et al., 2015); thus, AMF biotechnology in low-input planting systems such as organic cultivation is important in maintaining or improving the long-term soil fertility (Johansson et al., 2004). In recent years, the novel application of AMFs as biological fertilizers has improved the yield and quality of medicinal plants and stabilized the efficacy of these plants (Cheruth et al., 2009; Gianinazzi et al., 2010; Zeng et al., 2013). AMF was the most important factor affecting plant secondary pathways and metabolic (Spatafora et al., 2016). *S. miltiorrhiza* inoculation with AMFs promoted plant growth and increased contents of phenolic acid B (Sal B), tanshinone IIA (TS-IIA), and dihydrotanshinone I (DT-I) in *S. miltiorrhiza* roots (Wang et al., 2014; Li et al., 2016; Qi et al., 2016). Most studies have focused on commercial species, i.e., *Rhizophagus intraradices*, *Funneliformis mosseae*, and *Rh. irregularis.* while ignoring the rich diversity of native AMFs (Berruti et al., 2015). However, few studies have explored the effects of *S. miltiorrhiza* inoculation with native AMFs on the biomass, secondary metabolite content, and accumulation of roots.

In this study, eight native AMFs were isolated from the rhizosphere of semi-wild and farmland *S. miltiorrhiza*. As AMFs improved the yield and quality of medicinal plants, we evaluated whether these microorganisms affected *S. miltiorrhiza* roots in terms of biomass and active compound levels using physiological and phytochemical methods. The levels of the following metabolites were investigated: danshensu sodium (DS), caffeic acid (CA), rosmarinic acid (RA), Sal B, salvianolic acid A (Sal A), DT-I, cryptotanshinone (CT), tanshinone I (TS-I), and TS-IIA. We hypothesized that both biomass and active component levels in *S. miltiorrhiza* roots would respond positively to native AMF inoculation, to identify an ideal *S. miltiorrhiza* inoculum for Chinese medicine agriculture. Also, in considering the selectivity and functional diversity existing in AMF symbiosis (Helgason et al., 2002), we investigated the different responses of *S. miltiorrhiza* root to inoculants. Our results lay the foundation for screening symbiotic AMFs to improve the practical application of *S. miltiorrhiza*.

## Materials and Methods

### Experimental Setup and Design

The soil in this study was collected from “The base of *S. miltiorrhiza* in Meishan village” in Zhongjiang county, Sichuan province, Southwest China (30°57′6″N, 104°33′17″E). The sieved soil (pore size, 5 mm) was hermetically sterilized with 250 g⋅M^3^ dazomet for 1 week. A sterility test was negative before use. The soil was loam, with the following characteristics: pH 7.46; alkali-hydrolyzable nitrogen 128.93 mg⋅kg^–1^; available potassium (K) 43.50 mg⋅kg^–1^; available phosphorus (P) 34.71 mg⋅kg^–1^; and organic matter 35.1 mg⋅kg^–1^.

Test-tube seedlings were cultivated *in vitro*, whereas explants used the young leaves of *S. miltiorrhiza* Bge. cv. *sativa*. The medium for inducing proliferating plant was MS+2.0 mg⋅L^–1^ 6-BA+1.0 mg⋅L^–1^ NAA. Bud differentiation media was MS+1.0 mg⋅L^–1^ 6-BA+0.1 mg⋅L^–1^ NAA; the optimal rooting medium was 1/2 MS+0.2 mg⋅L^–1^ NAA+0.5 mg⋅L^–1^ IBA. The rooting rate was 94%. Rooted shoots (5 cm in length) were the source of pot culture plants for experiments.

Experiments were conducted in the greenhouse of the Chengdu University of Traditional Chinese Medicine, Chengdu, China. Pots (20 cm in diameter, and 30 cm in height) were autoclaved (121°C for 20 min) and filled with 3 kg sterilized soil. For each experimental pot, 400 spores of AMF species (20 g) were distributed around the roots of test-tube seedlings. Sterile water (121°C for 30 min) was then poured over plants. Plants used as non-mycorrhizal controls were not inoculated with AMF (hereafter referred to as NM). Plants were grown for 22 weeks in a glasshouse at 25∼30°C and relative humidity of 80% during the day and night. For the rest of the culture period, plants had natural temperature and humidity. Plants were watered 1/2 MS nutrient solution (without agar and sugar) once every 2 weeks during the greenhouse period.

**FIGURE 1:**
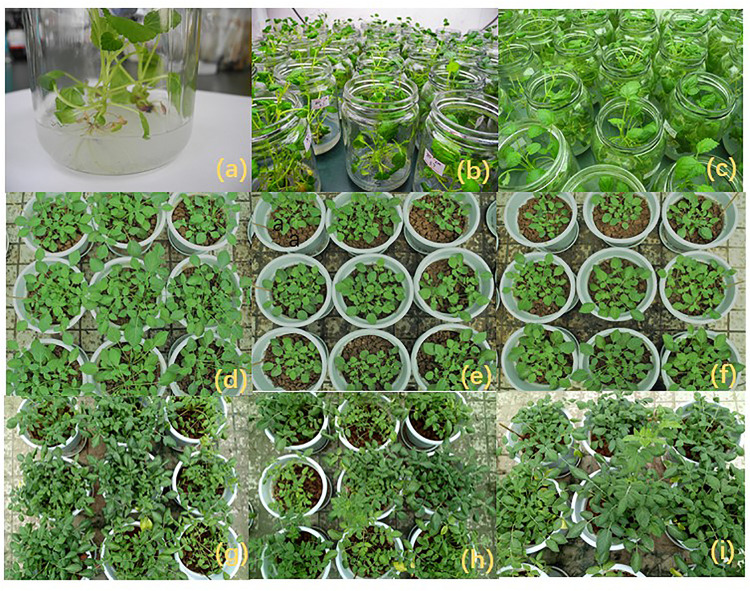
The growth of *S. miltiorrhiza* was is in good condition during the period of test-tube seedlings and potted. **(a–c)** The growth of rooted shoots (5 cm in length) which could be transplanted. (**d**–**f**) The growth of transplanted *S. miltiorrhiza* after 70 days. **(g–i)** The growth of *S. miltiorrhiza* after 150 days.

Pot experiments were generated as comparisons between the following nine treatments, as independent treatments arranged in a complete randomized design (Table 1). Each treatment had three independent replicates, equalling 27 pots in total. There were two *S. miltiorrhiza* plants in each pot (Figure 1).

**TABLE 1:** Effect of native AMFs inoculation on plant growth.

AMF species	FW (g)	DW (g)	Colonization (%)	MD (%)
GLF	41.53 ± 8.92	9.33 ± 2.96	73.30 ± 10.71	30.23
GLT	34.50 ± 8.63	6.10 ± 2.88	86.10 ± 1.68	–6.72
SEC	44.37 ± 3.43*	10.77 ± 2.56	60.52 ± 4.10	39.55
FUG	52.97 ± 17.51**	12.17 ± 3.44*	84.99 ± 2.76	46.51
RHM	36.43 ± 2.05	9.78 ± 0.15	54.83 ± 3.21	33.44
AMG	35.03 ± 3.49	9.85 ± 1.92	80.37 ± 2.83	33.91
ACL	21.77 ± 6.36	3.54 ± 1.56	69.77 ± 2.48	–83.90
ACT	39.67 ± 8.08	10.51 ± 2.17	79.75 ± 6.50	38.06
NM	28.67 ± 11.98	6.51 ± 3.17	–	−

*Means that there was a significant difference (p < 0.05) between the group compared with NM.

**Means that there was a highly significant difference (p < 0.01) between the group compared with NM. Data are mean ± SD (n = 6).

### Mycorrhizal Inoculants

Eight species of AMFs were used: Glomus formosanum (GLF), Gl. tenebrosum (GLT), Septoglomus constrictum (SEC), Funneliformis geosporum (FUG), Rhizophagus manihotis (RHM), Ambispora gerdemanii (AMG), Acaulospora laevis (ACL), Ac. tuberculata (ACT), isolated and preserved from rhizosphere soil samples of S. miltiorrhiza across 20 producing areas in China (Table 1; Liu et al., 2017). Spores from eight AMFs were isolated and propagated with Trifolium repens in pots containing sterilized soil and sand (1:1, v/v) (He et al., 2010). During the growth stage, each pot was watered with sterile water similar to field capacity, and pots received a 200 mL (pH = 6.0) nutrient solution (Hoagland and Arnon, 1938) every other week. The inoculum contained sand, soil, spores, external mycelium, and infected root fragments.

### Determination of Plant Growth and Mycorrhizal Colonization

After a growth period of 22 weeks, plants were harvested and divided into roots and shoots. The roots were separated, and weighed, and 2 g red roots were immersed in liquid nitrogen, and stored at −80°C for analyzed active ingredient investigations. Some roots were chopped into 1 cm long pieces and fixed in a formalin/acetic acid/ethanol (FAA, 13:5:200, v/v/v) solution for 24 h. The remaining roots were dried at 60°C and weighed to dry weight (DW) after constant weight at 105°C. The roots in the FAA were clarified and stained, using an acid fuchsin staining method (Dalpé and Séguin, 2013). Mycorrhizal colonization was estimated using an optical microscope (OLYMPUS CH20 BIMF200, Japan), by the presence or absence of fungal structures in the roots. The AMF infection rate was estimated using the following formula:

$$\mathrm{Percentage}\,\mathrm{of}\,\mathrm{root}\,\mathrm{colonization}\;=\frac{\mathrm{Total}\,\mathrm{number}\,\mathrm{of}\,\mathrm{infected}\,\mathrm{segments}\times 100}{\mathrm{Total}\,\mathrm{number}\,\mathrm{of}\,\mathrm{examined}\,\mathrm{root}\,\mathrm{segments}}$$

Mycorrhizal dependency (MD) was taken from the literature (Plenchette et al., 1983; Smith et al., 2003);

$$\text{MD}\left(\%\right)=\frac{\begin{array}{c} (\mathrm{dry}\mathrm{weight}\mathrm{of}\mathrm{mycorrhizal}\mathrm{plant}-\mathrm{average}\mathrm{dry}\\ \mathrm{weight}\mathrm{of}\text{non}-\text{mycorrhizalplant})\times 100 \end{array}}{\mathrm{dry}\,\mathrm{weight}\,\mathrm{of}\,\text{non}-\mathrm{mycorrhizal}\,\mathrm{plant}}$$

### UPLC Analysis

#### Sample Preparation

Frozen ground root tissue (0.5 g) was weighed and thawed in 10 mL 90% methanol, ultrasonic extracted for 45 min at 40°C, with the weight of the extracting solution made up by methanol. The sample was then centrifuged at 10,000 rpm for 10 min. The resulting supernatant was filtered through a 0.22 μm nylon membrane filter before injection into an ultra performance liquid chromatography (UPLC) system.

#### Active Ingredient Content Determination

**FIGURE 2:**
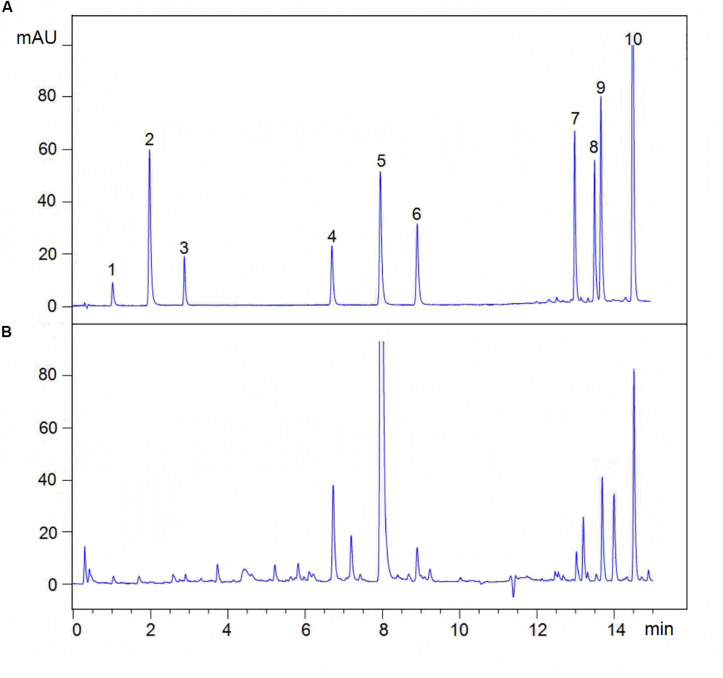
Typical UPLC chromatograms of **(A)** mixed standards and **(B)** sample. (1) Sodium danshensu, DS; (2) Protocatechuic aldehyde; (3) Caffeic acid, CA; (4) Rosmarinic acid, RA; (5) Salvianolic acid B, Sal B; (6) Salvianolic acid A, Sal A; (7) Dihydrotanshinone I, DT-I; (8) Tanshinone I, TS-I; (9) Cryptotanshinone, CT; (10) Tanshinone IA, TS-IIA.

Analyses were analyzed on an Agilent UPLC system (Agilent 1290, Agilent Corp.), including a binary solvent manager, sampler manager, column compartment, and photodiode array detector, connected to Agilent Infinity 2 software. The UPLC was fitted with an Agilent ZORBAX Eclipse Plus C18 column (2.1 × 50 mm, 1.8 μm). The column temperature was maintained at 35°C. Analyte separation was performed by using a gradient mobile phase consisting of 0.02% (v/v) phosphoric acid in water (A) and acetonitrile (B), modified from Li et al. (2014). Gradient condition was 0∼0.5 min, 95% A; 0.5∼2.0 min, 95∼87% A; 2.0∼6.5 min, 87∼78% A; 6.5∼10.0 min, 78∼72% A; 10.0∼11.5 min, 72∼40% A; 11.5∼15.0 min, 40∼10% A, and finally column reconditioning with 5% B isocratic for 2 min, after which the column was washed in 100% B for 3 min at a flow rate of 0.5 mL⋅min^–1^. The injection volume being 2 μL. The sample manager temperature was 10°C. The detection wavelength was 280 nm. Active ingredients identification was performed by comparing the retention times of samples with those of standards. Analytes above were quantified using the external standard method. The linear range, linear regression equations, and correlation coefficients of the standards curves are shown in Figure 2 and Table 2.

**TABLE 2:** The calibration curve data of reference substance in *S. miltiorrhiza* root (*n* = 6).

	Linear range (ng)	Regression equation	*r*^2^
DS	0.560∼8.960	y = 11.32557x−0.98662	0.99991
CA	0.168∼4.480	y = 56.20537x−6.85365	0.99996
RA	3.420∼68.400	y = 20.96961x−22.24801	0.99985
Sal B	91.60∼732.80	y = 6.62872x−44.93137	0.99997
Sal A	0.384∼10.240	y = 22.17195x−6.96602	0.99992
DT-I	0.204∼10.880	y = 58.09684x−10.34958	0.99984
TS-I	0.020∼0.672/	y = 72.97334x+1.66830	0.99990
CT	1.470∼78.400	y = 38.68225x−27.81167	0.99999
TS-IIA	0.730∼24.400	y = 63.73954x−29.18675	0.99997

### Data Calculation and Analysis

Mann-Whitney U tests were performed on treatment comparisons. Significant differences between treatments were confirmed by multiple comparisons by the Mann-Whitney U method at a 5% significance level. Statistical analyses were performed using IBM SPSS 21.0 for Windows (IBM Corp, Armonk, NY, United States).

Similarities in sample chromatographic patterns were evaluated using “Chromatogram Analysis and Data Management System for Traditional Chinese medicine” (ver. 2012). Principal component analysis (PCA) and hierarchical cluster analysis (HCA) were conducted with origin 2019 for Windows (OriginLab, Hampton, Massachusetts, United States). All dates were normalized into Z-scores before analysis.

## Results

### AM Colonization

Percentage of colonization indices were used for AMF activities (Smith and Smith, 2011). All eight AMFs showed full colonization in inoculated plants after 22 weeks of growth, with a colonization rate of more than 54.83 ± 3.21%, of which GLT colonization rate was the highest (86.10 ± 1.68%) (Table 1). The percentage of hyphae colonization varied among species (GLT > FUG > AMG > ACT > GLF > ACL > SEC > RHM).

### Root Biomass Measurements

*S. miltiorrhiza* root weights were greatly affected by symbiosis (Table 1). However, the fresh root weight (FW) of *S. miltiorrhiza* colonized by ACL was decreased, and so did dry weight of GLF-plant and ACL-plant, whereas the rest of the plants were increased. Of these, plants inoculated with FUG or SEC significantly increased the FW and DW of roots to 55, 85, 65, and 87%, respectively. *S. miltiorrhiza* plants exhibited different mycorrhizal dependencies (MDs) for different AMFs, with large differences (range, i.e., −83.9–46.51%).

### Active Ingredients Content and Composition

The contents of nine secondary metabolites (DS, CA, RA, Sal B, Sal A, DT-I, CT, TS-I, and TS-IIA) in each sample were calculated from standard curves (Tables 2, 3). When compared with NM, most AMF-plants exhibited promoting effects on the content of nine compounds, especially phenolic acids. Total phenolic acids (TP) were reduced by FUG (*p* < 0.05) and ALC treatments, whereas the others showed a promotion range, i.e., 8.02–44.07%. Plants inoculated with GLF significantly increased the contents of TP (*p* < 0.01). When compared with phenolic acids, inoculation treatment had limited effects on total tanshinones (TTS). *S. miltiorrhiza* inoculation with GLT, SEC, FUG, and ACT variably increased TTS levels, but the remaining groups had no positive effects.

**TABLE 3:** Active ingredients concentration (values are expressed on a dry weight basis) rate in *S. miltiorrhiza* root (mg⋅g^–1^ DW).

Sp.	DS	CA	RA	Sal B	Sal A	DT-1	TS-1	CT	TS-IIA	TP	TTS
GLF	0.14 ± 0.03	0.06 ± 0.03	4.15 ± 0.50*	45.01 ± 3.90**	0.44 ± 0.03**	0.05 ± 0.03	0.009 ± 0.002	0.88 ± 0.62	0.59 ± 0.33	49.79 ± 4.32**	1.52 ± 0.98
GLT	0.11 ± 0.02	0.09 ± 0.03	3.07 ± 1.08	33.70 ± 6.91	0.37 ± 0.07	0.12 ± 0.04	0.007 ± 0.003	1.74 ± 0.84	0.76 ± 0.31	37.33 ± 7.89	2.63 ± 1.18
SEC	0.13 ± 0.03	0.04 ± 0.00	3.22 ± 1.01	36.40 ± 4.81	0.33 ± 0.04	0.04 ± 0.02	0.008 ± 0.006	1.04 ± 1.05	0.59 ± 0.47	40.12 ± 5.76	1.67 ± 1.55
FUG	0.05 ± 0.00**	0.06 ± 0.02	1.03 ± 0.55**	18.41 ± 6.35*	0.13 ± 0.07*	0.06 ± 0.02	0.011 ± 0.001	1.33 ± 0.30	0.95 ± 0.18	19.68 ± 6.92*	2.36 ± 0.28
RHM	0.08 ± 0.02*	0.04 ± 0.00	3.31 ± 0.68	38.90 ± 5.86	0.33 ± 0.07	0.03 ± 0.01	0.008 ± 0.001	0.52 ± 0.12	0.41 ± 0.09	42.66 ± 6.61	0.97 ± 0.2
AMG	0.11 ± 0.05	0.04 ± 0.01	2.44 ± 0.54	36.64 ± 2.01*	0.30 ± 0.05	0.03 ± 0.00	0.007 ± 0.003	0.77 ± 0.24	0.53 ± 0.18	39.52 ± 1.92	1.33 ± 0.41
ACL	0.15 ± 0.05	0.07 ± 0.03	4.76 ± 0.42**	41.23 ± 8.27	0.45 ± 0.12	0.06 ± 0.01	0.013 ± 0.002*	0.71 ± 0.14	0.43 ± 0.13	46.66 ± 8.72	1.21 ± 0.28
ACT	0.09 ± 0.01	0.02 ± 0.01	2.73 ± 0.19	30.23 ± 2.35	0.36 ± 0.04	0.06 ± 0.02	0.011 ± 0.003	1.17 ± 0.31	0.64 ± 0.19	33.43 ± 2.49	1.88 ± 0.49
NM	0.12 ± 0.02	0.04 ± 0.02	3.34 ± 0.75	30.75 ± 3.00	0.31 ± 0.03	0.05 ± 0.02	0.007 ± 0.003	0.94 ± 0.59	0.53 ± 0.47	34.56 ± 3.77	1.53 ± 1.04

*Means that there was a significant difference (p < 0.05) between the group compared with NM.

**Means that there was highly significant difference (p < 0.01) between the group compared with NM. Data are mean ± SD (n = 6).

To explore compositional changes in *S. miltiorrhiza* active ingredients, we explored the active ingredient molar content (which is equal to the content of each active ingredient divided by the respective molar mass; Table 4). The results showed that different strains had different effects on active ingredients molar contents in *S. miltiorrhiza*. But overall, the impact was greatest on phenolic acids, especially Sal B. The molar content of Sal B was increased when inoculated with GLF (*p* < 0.01), GLT, SEC, RHM, and AMG (*p* < 0.05), but upon FUG inoculation, a significant reduction (*p* < 0.01). In contrast to FUG colonization, GLF, AMG, and ACL colonization increased TP molar content. Thus, most treatment groups increased TP molar content, whereas FUG and ACT reduced this level.

**TABLE 4:** Active ingredients molar content (values are expressed on a dry weight basis) in *S. miltiorrhiza* root (mol⋅g^–1^ DW).

Sp.	DS	CA	RA	Sal B	Sal A	DT-1	TS-1	CT	TS-IIA	TP	TTS
GLF	0.63 ± 0.15	0.32 ± 0.16	11.52 ± 1.38	62.68 ± 5.43**	0.88 ± 0.06**	0.17 ± 0.09	0.03 ± 0.01	2.96 ± 2.1	2.01 ± 1.12	76.04 ± 6.74**	5.17 ± 3.31
GLT	0.5 ± 0.1	0.48 ± 0.18*	8.53 ± 3	46.93 ± 9.62	0.75 ± 0.15	0.42 ± 0.14**	0.03 ± 0.01	5.89 ± 2.82	2.6 ± 1.07	57.17 ± 12.37	8.93 ± 4.02
SEC	0.6 ± 0.12	0.23 ± 0.01	8.94 ± 2.84	50.68 ± 6.7	0.67 ± 0.08	0.13 ± 0.06	0.03 ± 0.02	3.51 ± 3.58	2 ± 1.62	61.13 ± 9.33	5.67 ± 5.24
FUG	0.22 ± 0.01**	0.34 ± 0.08	2.85 ± 1.51**	25.64 ± 8.84**	0.27 ± 0.14**	0.22 ± 0.08	0.04 ± 0.01**	4.5 ± 1.02	3.24 ± 0.6	29.32 ± 10.35**	8 ± 0.96
RHM	0.34 ± 0.08*	0.22 ± 0.02	9.2 ± 1.9	54.17 ± 8.16*	0.67 ± 0.14	0.12 ± 0.03	0.03 ± 0	1.75 ± 0.4	1.4 ± 0.3	64.6 ± 10.24	3.3 ± 0.67
AMG	0.48 ± 0.23	0.22 ± 0.06	6.77 ± 1.51*	51.02 ± 2.79**	0.6 ± 0.1	0.11 ± 0.01	0.02 ± 0.01	2.58 ± 0.81	1.8 ± 0.62	59.09 ± 2.82	4.52 ± 1.39
ACL	0.67 ± 0.23	0.41 ± 0.14*	13.23 ± 1.18*	57.41 ± 11.52*	0.9 ± 0.24*	0.21 ± 0.03	0.05 ± 0.01**	2.39 ± 0.49	1.45 ± 0.45	72.62 ± 12.79*	4.1 ± 0.94
ACT	0.39 ± 0.06**	0.13 ± 0.03	7.58 ± 0.53	42.09 ± 3.27	0.72 ± 0.08	0.21 ± 0.08	0.04 ± 0.01**	3.95 ± 1.05	2.17 ± 0.64	50.92 ± 3.68	6.36 ± 1.67
NM	0.56 ± 0.1	0.21 ± 0.09	9.28 ± 2.07	42.82 ± 4.17	0.62 ± 0.06	0.17 ± 0.07	0.02 ± 0.01	3.18 ± 2.01	1.8 ± 1.6	53.49 ± 6.4	5.18 ± 3.53

*Means that there was a significant difference (p < 0.05) between the group compared with NM.

**Means that there was highly significant difference (p < 0.01) between the group compared with NM. Data are mean ± SD (n = 6).

In brief, the composition and content of the nine compounds in *S. miltiorrhiza* roots were variably affected by the eight AMFs, especially phenolic acids. However, FUG treatment significantly reduced TP content, whereas other treatments increased these levels.

### Active Ingredient Accumulation

The accumulation of active ingredients (content × DW) per plant was used as the most common index for medical plant productivity (Zhao et al., 2015). Most AMF treatments promoted the accumulation of active ingredients from *S. miltiorrhiza* individual roots, especially phenolic acid (Table 5). GLF treatment promoted TP accumulation in plants, which reached the maximum accumulation of 227.85 mg/plant (*p* < 0.01). Equally, SEC, RHM and AMG treatments significantly promoted Sal B accumulation, which increased by 81.24∼91.93% (*p* < 0.05). Although inoculation with GLT or FUG also exerted promotional effects on TP, the effect was small (*p* > 0.05). Additionally, we observed that ACL-treated plants exerted some inhibitory effects on both TP and TTS components.

**TABLE 5:** The accumulation of active ingredients per plants from *S. miltiorrhiza* root (mg).

Sp.	DS	CA	RA	Sal B	Sal A	DT-1	TS-1	CT	TS-IIA	TP	TTS
GLF	0.62 ± 0.05	0.24 ± 0.06	19.12 ± 5.32*	205.85 ± 46.04**	2.03 ± 0.69**	0.21 ± 0.12	0.039 ± 0.013	3.97 ± 2.96	2.67 ± 1.56	227.86 ± 51.93**	6.89 ± 4.65
GLT	0.35 ± 0.2	0.23 ± 0.03	9.2 ± 3.99	105.15 ± 56.73	1.16 ± 0.66	0.31 ± 0.04	0.024 ± 0.015	4.43 ± 1.09	1.99 ± 0.28	116.08 ± 61.43	6.75 ± 1.36
SEC	0.72 ± 0.24*	0.22 ± 0.05	16.38 ± 2.31	192.05 ± 23.63*	1.74 ± 0.23*	0.2 ± 0.13	0.043 ± 0.039	6.48 ± 8.43	3.52 ± 3.89	211.12 ± 21.69*	10.25 ± 12.45
FUG	0.3 ± 0.08	0.37 ± 0.17	5.86 ± 3.11	107.2 ± 41.38	0.79 ± 0.53	0.34 ± 0.03	0.065 ± 0.021	7.73 ± 0.73	5.89 ± 2.49	114.53 ± 44.86	14.02 ± 2.76
RHM	0.37 ± 0.09	0.19 ± 0.01	16.21 ± 3.73	190.31 ± 32.7*	1.62 ± 0.36	0.16 ± 0.04	0.038 ± 0.002	2.54 ± 0.64	2.02 ± 0.49	208.7 ± 36.83*	4.76 ± 1.08
AMG	0.49 ± 0.16	0.19 ± 0.04	11.67 ± 1.13	181.35 ± 43.01*	1.44 ± 0.2	0.15 ± 0.01	0.032 ± 0.01	3.91 ± 1.94	2.73 ± 1.54	195.14 ± 42.89*	6.83 ± 3.47
ACL	0.24 ± 0.1	0.12 ± 0.01	8.29 ± 3.3	72.07 ± 35.47	0.76 ± 0.36	0.1 ± 0.04	0.023 ± 0.013	1.2 ± 0.46	0.74 ± 0.38	81.49 ± 39.05	2.06 ± 0.88
ACT	0.46 ± 0.13	0.12 ± 0.03	14.38 ± 3.26	157.57 ± 22.94	1.86 ± 0.27*	0.32 ± 0.21	0.061 ± 0.027	6.34 ± 3.09	3.4 ± 1.45	174.38 ± 26.54	10.12 ± 4.68
NM	0.38 ± 0.14	0.12 ± 0.05	10.68 ± 5.2	100.06 ± 50.61	0.97 ± 0.39	0.15 ± 0.09	0.023 ± 0.019	3.62 ± 3.95	2.27 ± 3.01	112.22 ± 56.28	6.06 ± 7.03

*Means that there was a significant difference (p < 0.05) between the group compared with NM.

**Means that there was highly significant difference (p < 0.01) between the group compared with NM. Data are mean ± SD (n = 6).

**FIGURE 3:**
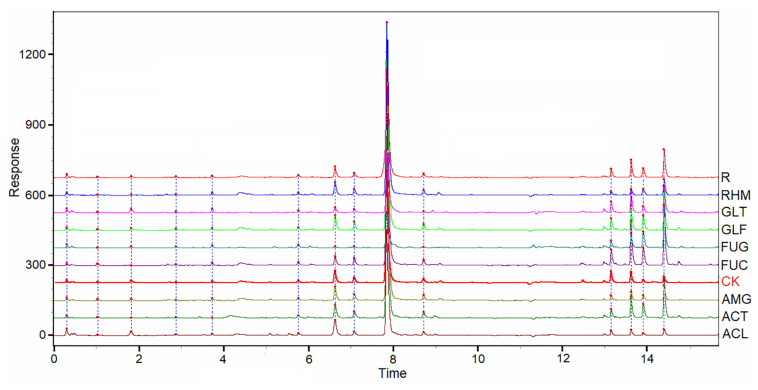
UPLC for partial samples of *S. miltiorrhiza* root and the simulative mean chromatogram (R).

The chromatographic patterns from 54 samples of *S. miltiorrhiza* root samples were evaluated systematically using “Chromatogram Analysis and Data Management System for Traditional Chinese Medicine” (ver. 2012). The correlation coefficient of each chromatogram to the simulative mean chromatogram was 0.974 ± 0.06 (mean ± *SD*). However, differences in the chemical characteristics were identified in *S. miltiorrhiza* plants inoculated with eight AMF species, when compared with those in NM plants. These chromatograms are shown (Figure 3). The correlation coefficients of eight chromatograms of AMF-plant samples of the NM plants were 0.998 (ACL), 0.998 (AMG), 0.996 (RHM), 0.992 (GLF), 0.989 (ACT), 0.972 (SEC), 0.954 (GLT), and 0.758 (FUG), respectively. The correlation coefficient of FUG group was different from other groups, which was in line with the results above (Tables 1, 3).

**FIGURE 4:**
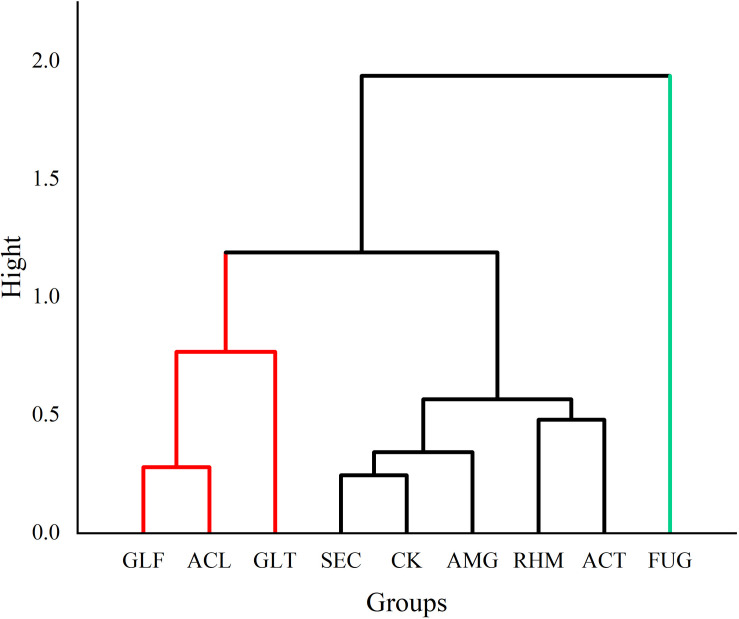
Score chart of PCA of different AMF treatment groups (phenolic acids).

**FIGURE 5:**
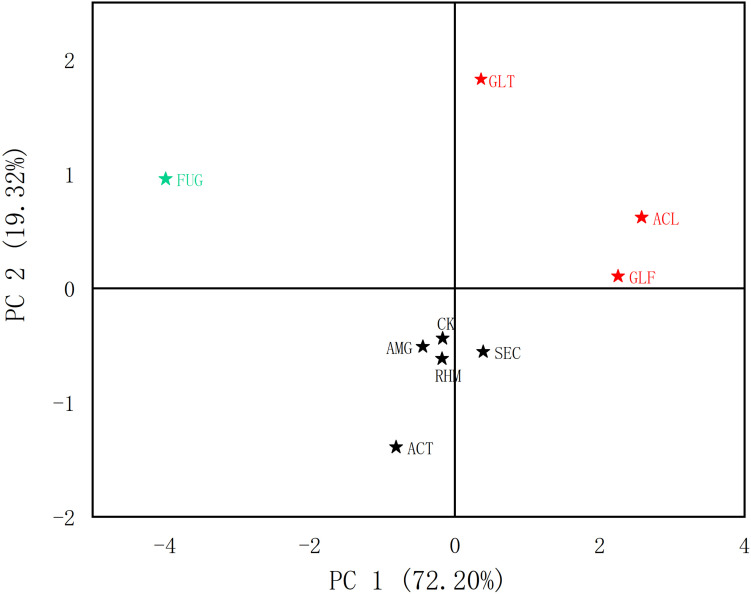
Diagram of HCA of different AMF treatment groups (phenolic acids).

From our quantitative data on phenolic acid content, we observed differences. PCA was used to analyze differences between plant roots to explore the relationship between compounds and sample clustering. The results in Figure 4 showed that samples relating to the first two principal components (PCs) had a good tendency to separate (91.52% of the total variance). As shown (Figure 5), samples were divided into three groups: (1) FUG-plants exerted significant inhibitory effects on phenolic acid content. (2) Plant inoculation with GLF and ACL significantly promote phenolic acid content. (3) The remaining groups had variable promotional promotion effects on phenolic acid levels, consistent with Table 3. As shown (Figure 5), HCA analysis also generated similar results.

## Discussion

In this study, we determined the effects of eight native AMFs colonization on growth and secondary metabolite production of *S. miltiorrhiza* roots. As a traditional and celebrated Chinese root medicine, *S. miltiorrhiza* was paid more attention to the changes in the biomass and secondary metabolites of its roots. The premise of this research on the influence of AMF symbiosis on *S. miltiorrhiza* was mainly the application of genetically homogeneous sterile test tube vaccines. Most research studies currently mainly used seeds, while seeds usually carried bacteria, callus was selected to generate sterile test tube seedlings to control experiments and observe symbiosis between plants and AMFs. A high degree of functional diversity exists among AMF species, which affects plants’ response (Kapoor et al., 2007). Therefore, we sought to explore *S. miltiorrhiza* response to colonization of different native AMFs. We also explored the influence of AMF colonization on biomass and secondary metabolite production of the plant roots.

### Influence of Mycorrhizal Formation

Mycorrhiza is a mutually beneficial symbiosis formed between soil fungi (AMFs) and plant roots. AMFs help plants capture water and mineral nutrients (especially P) from the soil, and in return, approximately 20% of plant-fixed carbon was transferred to fungi (Smith et al., 2003). According to soil nutrient classification standard from the second national soil census, the tested soils were slightly enriched in organic matter (grade II, 30–40 g⋅kg^–1^), alkalic nitrogen (grade II, 120–150 g⋅kg^–1^), and available P (grade II, 20–40 mg⋅kg^–1^). In our study, the 1/2MS culture solution (no sugar and agar) was facilitated plant growth without visible deficiencies. It is assumed that the nutrient content such as nitrogen and P in cultivation test soils is sufficient and satisfying plant growth (Jiang and Wei, 2004; Qi et al., 2016). As a mycotrophic plant, *S. miltiorrhiza* is easily colonized by AMFs in nature or experiment study to form good colonization rates (Yang et al., 2017). Equally, when compared with commercial species, native species tend to be more infectious and quickly colonize host plants roots (Chenchouni et al., 2020). In our study, the colonization rate was more than 54.83 ± 3.21%, indicating AMFs had high affinities with the host *S. miltiorrhiza* (Table 1). Our study also showed that AMF colonization intensities depended on the species, and varied from 54.83 to 86.10%; these percentages were influenced by fungal characteristics, reproduction density, soil properties, plant genes, and P supply (Smith and Smith, 2012; Zubek et al., 2012b).

### Influence of Plant Biomass

Our study only evaluated the underground medicinal parts (roots) of *S. miltiorrhiza*, because the above ground parts (shoots) of some plants had withered at the time of harvest. The results showed that the eight native AMFs exerted different effects on S. *miltiorrhiza* growth under greenhouse conditions (Table 1). In general, AMF inoculation induced increased biomass of *S. miltiorrhiza* roots, consistent with previous studies (Hoeksema et al., 2010; Shi et al., 2016; Tarraf et al., 2017; Chenchouni et al., 2020). Another study showed that plant biomass after AMF colonization was 3.1 times higher than that of unvaccinated plants on average (Hoeksema et al., 2010). These increased biomass data, induced by mycorrhizal symbiosis, may be generated by large numbers of fungal hyphae, increasing the root absorption surface areas, thereby promoting the plant’s ability to absorb and assimilate nutrients (Bowles et al., 2016). Positive growth responses to AMF colonization were usually attributed to attributable to increased P uptake by fungi, thereby alleviating P deficiency and promoting growth. The migration rate of P in the soil is generally low, thus, direct absorption by plants via root hairs forms depletion zones around roots (Schachtman et al., 1998), potentially limiting further absorption. The extra-root hyphae from AMFs could enter soil pores undetected by the root system and potentially extended to the soil space by 25 cm from the root system to drive nutrients (Jansa et al., 2003). This greatly improves the nutrient absorption, and utilization, and translocation capabilities (Bowles et al., 2016), and leads to an increase in photosynthetic product yields and plant biomass.

Previous research that AMF had high functional diversity during *S. miltiorrhiza* colonization, even the same AMF genus exerted highly variable effects on plant growth (Kapoor et al., 2007; Thonar et al., 2011). In agreement, when our study plants were inoculated with ACL and ACT, respectively; the FW (21.77 ± 6.36) and DW (3.54 ± 1.5) of ACL-plants were significantly lower than those of the NM group, while ACT-plants was significantly higher than the NM group, i.e., 39.67 ± 8.08 and 10.51 ± 2.17, respectively. Plant productivity depends on photosynthesis, and this process depends on P-containing compounds. The measurement of P content and utilization efficiency in plant organs or branches biomass could more intuitively explain this high variability. Therefore, in view of AMF functional heterogeneity, screening suitable host AMF strains is crucial for improving *S*. *miltiorrhiza* productivity. In some cases, fungal symbiosis may also be detrimental to plant growth. For example, root FW and DW of plants inoculated with ACL or GLT were decreased (Table 1). One reason could be that when plants and fungi compete for limited nutrients, fungi sometimes isolate nutrients in their tissues, fail to pass these onto plants, while consuming the photosynthetic products of plants (Lerat et al., 2003). Equally, internal changes in plants caused by AMF colonization may reduce plant nutrients via systemic changes in phytochemistry or gene expression (Hodge et al., 2010).

As an index to measure the influence of AMF inoculation on plant growth and development, MD could characterize plant growth and development (Tawaraya, 2003). ACL-plants inhibited plant growth and had the lowest MD value (−83.9%), while FUG-plant significantly promoted plant growth and had the highest MD (46.51%). Therefore, these data indicated that most AMF inoculations with *S. miltiorrhiza* had variable growth and development effect, thus, selecting optimized AMF treated with *S. miltiorrhiza* could improve symbiosis and growth (Rengel, 2002).

### Effects of Secondary Metabolites

Plant secondary metabolites are compounds that are not required for basic metabolism, but they are essential for the plant interactions with the environment. The composition and content of secondary metabolites are affected by biological, non-biological, and agronomic management factors. AMFs play important roles in these processes; they improve plant nutrition and health, induce changes in metabolic levels, and promote metabolite synthesis (Sbrana et al., 2014).

Mycorrhization not only affects plant growth but also affected plant secondary metabolism, sometimes eliciting positive changes medicinal compounds (Rapparini et al., 2007; Chaudhary et al., 2008). Many studies have explored the impact of AMF colonization on secondary metabolites in host medicinal plants, e.g., *Artemisia annua* L. (Domokos et al., 2018), *Valeriana officinalis* L. (Nell et al., 2010), *Anadenanthera colubrina* (Vell.) Brenan (Kapoor et al., 2007), *Cynara cardunculus* L. var. *scolymus* (Ceccarelli et al., 2010), yam (*Dioscorea* spp.) (Lu et al., 2015), *Angelica archangelica* L. (Zitterl-Eglseer et al., 2015), *Ocimum basilicum* (Toussaint et al., 2007), *Panax quinquefolius* L. (Smith et al., 2010). In terms of AMF-plant symbiosis, secondary metabolites have been shown to undergo quantitative changes, usually responded to the host plant to AMF inoculation (Pozo and Azcón-Aguilar, 2007). Generally speaking, compounds such as phenols and terpenoids, are usually produced by host plants to resist infection by foreign microorganisms (Zhang et al., 2015).

In our study, AMF inoculations of *S. miltiorrhiza* had a greater impact on phenolic acids, when compared to tanshinones. *S. miltiorrhiza* inoculation with GLF, AMG, and ACL significantly increased phenolic acid levels (*p* < 0.05). Previous studies have shown that Phenolic acids are the most studied components affected by mycorrhizal colonization (Schaarschmidt et al., 2007; López-Ráez et al., 2010), and they are also important plant secondary metabolites for plant defense (Yao et al., 2007). AMF increased the phenolic synthesis in roots probably via signaling pathways of hydrogen peroxide, salicylic acid, and nitric oxide in a signaling cascade (Zhang et al., 2013).

Although plant inoculation with FUG significantly inhibited salvianolic acid production, it exerted strong promoting effects on tanshinones components. Equally, we observed that FW (52.97 ± 17.51) and DW (12.17 ± 3.44) of FUG-plant were significantly higher than those of the NM group, which resulting in the accumulation of tanshinone components of FUG-plant particularly prominent. Welling et al. (2016) observed that terpenoids accumulation in plants colonized by AMFs might be related to changes in plant morphology, P utilization, and gene transcription in terpenoid biosynthesis pathways. In addition, terpenoids are defensive mechanisms against herbivores and pathogens, but act as signal and reward molecules to beneficial organisms (e.g., mycorrhiza) (Pichersky and Raguso, 2016).

In *S. miltiorrhiza* plants inoculated with AMFs, changes in the accumulation of active ingredient levels were inconsistent, but some differences were significant. TP contents of GLF and ACL plants were significantly higher than those in NM-plant, but TTS levels were slightly lower than those in NM-plants. However, GLT- and ACT plants, which had the same genus, showed variable promotional effects toward TP and TTS, but no significant differences were observed. In summary, our results indicated that secondary metabolites contents differed between different AMF treatments. Therefore, it was important to select effective AMF species to improve *S. miltiorrhiza* quality.

At present, the production of higher concentrations of secondary metabolites had higher practical application value, such as in the physical therapy (Zubek et al., 2012a) and the food industries (Eftekhari et al., 2012). Hundreds of secondary metabolites are contained in *S. miltiorrhiza* roots; the main active ingredients are DS, CA, RA, Sal B, Sal A, DT-I, CT, TS-I, and TS-IIA (Wan et al., 2020). These are some of the main components extracted by the pharmaceutical industry. Our study showed that AMFs affected bioaccumulation yields of the effective components of *S. miltiorrhiza* roots. We observed significant differences in active ingredients accumulation in *S. miltiorrhiza* roots with different native AMF treatments. Apart from ACL, AMFs inoculation showed consistent promotional effects on active ingredients accumulation in *S. miltiorrhiza* roots. Similarly, accumulation of phenolic acid and tanshinones were increased by 102.92 and 131.35%, respectively. Similar to secondary metabolite contents, the amount of accumulation varied significantly among the same genus, e.g., ACL and ACT plants. The accumulation of active ingredients is economically significant for effective AMF selection.

### The Relationship Between AMF Isolation Frequencies and Host Growth, and Secondary Metabolism

The AMF isolation frequency [IF = (the number of soil samples in AMF/total soil samples) × 100%] reveals the distribution probability of native AMFs (Liu and Chen, 2007). In this study, eight native AMF species collected from 20 rice planting bases in China were selected (Liu et al., 2017). The IF at each base was ACL (90%), RHM (80%), ACT (75%), AMG (40%), FUG (35%), GLF (10%), GLT (5%), and SEC (5%). We generally believed that widely distributed species increased plant growth and biomass. For example, *Fu. mosseae* and *Rh. intraradices* were most widely distributed in the root system of *Olea europaea* L. (Kong et al., 2019), and significantly increased the number of lateral branches and leaves, total phenolic chlorophyll, and carotenoids content in the leaves of seedlings (Seifi et al., 2014). Cassava (*Manihot esculenta* Crantz) inoculation with *Ac. colombiana*, the most abundant of a native AMF, significantly increased crop yields (Séry et al., 2016). Our study showed that widely distributed species (IF > 70%, ACT and RHM) promoted the accumulation of plant biomass (>27%), whereas ACL, as the most widely distributed species (IF > 90%), decreased plant biomass. Spices with lower distribution frequencies (IF < 10%) had variable effects on plant biomass; when compared with that of NM-plants, the DW of SEC-plants increased by 65%, while that of GLT-plants decreased by 6%. Equally, we also observed that inoculation of *S. miltiorrhiza* with middle distribution frequency species, i.e., GLF, FUG, and AMG, promoted plant biomass accumulation.

Similar to plant biomass effects, AMFs with different IFs altered plant secondary metabolites. Species with high distribution frequencies, RHM and ACL increased phenolic acid contents and decreased tanshinones. In contrast, ACT increased tanshinones contents and decreased phenolic acids. The medium distribution frequency fungi groups, i.e., GLF, FUG, and AMG, also showed similar patterns. GLF and AMG promoted TP, whereas FUG significantly inhibited TP. Although the distribution frequency of GLT and SEC was low (IF < 10%), both increased phenolic acids and tanshinones. This is a bit different from the studies on the synthesis and accumulation of secondary metabolites of *Gl. mosseae* inoculated with medicinal plants (Wang et al., 2020), such as *Atractylodes macrocephala* Koidz. (Wang et al., 2010), *Astragalus membranaceus* var. *mongholicus*, and *Artemisia annua* L. (Tan et al., 2013). So, considering the accumulation of biomass and metabolites in *S. miltiorrhiza* plants, we believe AMFs with a low IF is more beneficial to plant growth and secondary metabolites accumulation.

### Application Prospects of AMF in *S. miltiorrhiza*

The symbiotic relationship between AMFs and plants was practically ubiquitous across terrestrial ecosystems (Finlay, 2008). AMFs are considered to be natural biological fertilizers with high ecological significance. They provide water, nutrients, and pathogen protection for the hosts while they exchange photosynthetic products, and improve the host productivity and resistance to nutritional stress (Feddermann et al., 2010; Begum et al., 2019). It is generally believed that AMFs could act as substitutes for inorganic fertilizers because the application of biological fertilizers could effectively reduce the quantitative use of chemical fertilizer inputs in the near future, especially the amount of P (Ortas, 2012). Although AMFs have great potential, they have not been fully adopted by farmers (Ryan and Graham, 2018). However, to achieve sustainable ecological agriculture of traditional Chinese medicines, it is important to establish a natural level of AMF richness, and it was an effective alternative to using traditional fertilization to improve the quality and yield of *S. miltiorrhiza*.

The study showed that AMFs and *S. miltiorrhiza* formed good symbiotic relationships, but different symbiotic affinity performance was observed. Medicinal material yields and active ingredient contents were important for evaluating the beneficial effects of AMFs on medicinal plants. We observed different AMFs exerted different effects on the biomass and secondary metabolite levels in plants. Therefore, our comprehensive evaluation of AMF treatments could influence the future ecological planting of *S. miltiorrhiza*.

*S. miltiorrhiza* inoculation with GLF promoted roots biomass accumulation and significantly increased the content and accumulation of TP, 44.07 and 102.92%, respectively. However, TTS content and accumulation in GLF-plants generated limited changes. Therefore, we believe GLF inoculation was effective in generating phenolic acid compounds in *S. miltiorrhiza*.

When compared with that of the NM group, the inoculation of *S. miltiorrhiza* with FUG significantly increased the yield of roots by 84.76% (FW, *p* < 0.01) and 86.95% (DW, *p* < 0.05), respectively. Although the TP levels of FUG-plant roots were significantly reduced, the content and accumulation of TTS were increased by 54.25 and 131.35%, respectively. Therefore, we believe that SEC colonization of *S. miltiorrhiza* effectively generated high levels of TTS compounds in *S. miltiorrhiza.*

Not only was beneficial to plant growth, but also effectively increased TTS and TP content and accumulation, which was agreed with our comprehensive evaluation of medicinal materials. Therefore, we propose a reliable method for improving the yield and quality of *S. miltiorrhiza* raw materials by inoculating with SEC. Our data suggested that mycorrhizal symbiosis may be used as a biotechnological method for the production effects of phenolic acid compounds in medicinal materials. GLF, FUG, and SEC species were suitable for planting *S. miltiorrhiza* biological fertilizers, which could increase the yield and quality of medicinal materials. Although the accumulation mechanism of secondary metabolites mediated by AMFs, however, they are still unclear.

## Conclusion

In conclusion, the results showed that *S. miltiorrhiza* formed a good symbiotic relationship with a variety of native AMFs. *S. miltiorrhiza* inoculation with AMFs promoted the accumulation of plant biomass and secondary metabolites (especially phenolic acids), although changes were not always positive. In addition, native AMF species exhibited high functional specialization, even in the same genus. Different AMF species showed different effects on biomass and secondary metabolite accumulation in *S. miltiorrhiza*. Therefore, the results contribute to select effective AMF species to inoculate *S. miltiorrhiza* to generate optimized planting effects for ecological planting. Equally, when compared with widely distributed species, narrower distributed species shad a stronger promotional effect on plant growth and secondary metabolism. In summary, native AMF inoculation with *S. miltiorrhiza* promoted the growth of the roots of the plant and increased the levels of secondary metabolites. Thus, native AMFs have huge potential in the ecological cultivation and production of *S. miltiorrhiza*.

## Data Availability Statement

The raw data supporting the conclusions of this article will be made available by the authors, without undue reservation.

## Author Contributions

Z-YY conceived and designed the experiments. Y-HW and HW was responsible for preparing the first draft of the manuscript. ML and BL revised the manuscript. All authors performed the experiments. All authors contributed to the article and approved the submitted version.

## Conflict of Interest

The authors declare that the research was conducted in the absence of any commercial or financial relationships that could be construed as a potential conflict of interest.

## Abbreviations

AMF: arbuscular mycorrhiza fungi
Sal B: salvianolic acid B
TS-IIA: tanshinone IIA
DT-I: dihydrotanshinone I
DS: danshensu
CA: caffeic acid
RA: rosmarinic acid
Sal A: salvianolic acid A
CT: cryptotanshinone
TS-I: Tanshinone I
MD: mycorrhizal dependency
FW: fresh weight
DW: dry weight
TP: total phenolic acids
TTS: total tanshinones
PCA: principal component analysis
HCA: hierarchical cluster analysis
IF: isolation frequency.
